# Biogenic synthesis of silver nanoparticles (AgNPs) from *Allium jacquemontii* extract and its assessment in different biological activities

**DOI:** 10.1038/s41598-025-10828-y

**Published:** 2025-07-29

**Authors:** Asif Kamal, Moona Nazish, Tehmina Siddique, Muhammad Azhar Khan, Narjis Khatoon, Ghulam Mujtaba Shah, Jawaher Alzahrani, Wasim Akhtar, Shoaib Noor, Muhammad Tahir Naseem, Wajid Zaman

**Affiliations:** 1 Department of Plant Sciences, Faculty of Biological Sciences, Quaid-I-Azam University , Islamabad, 45320 Pakistan; 2 Govt. Sadiq College Women University, Bahawalpur, Punjab Pakistan; 3 Department of Botany, Hazara University, Mansehra, 21300 KPK Pakistan; 4 Center of Agricultural Biochemistry and Biotechnology, University of Agriculture, Faisalabad, Punjab Pakistan; 5 Department of Zoology, College of Science, King Saud University, 11451 Riyadh, Saudi Arabia; 6 Department of Botany, University of Azad Jammu and Kashmir, Muzaffarabad, 13100 Pakistan; 7 Department of Electronic Engineering, Yeungnam University, Gyeongsan, 38541 Republic of Korea; 8 Department of Life Sciences, Yeungnam University, Gyeongsan, 38541, Republic of Korea

**Keywords:** Nanotechnology, Silver nanoparticles (AgNPs), *Allium jacquemontii*, HPLC analysis, Biological activities, Biotechnology, Plant sciences

## Abstract

Nanotechnology is a vibrant and fast-developing field in science with diverse applications. Silver nanoparticles (AgNPs) are becoming gradually valuable because of their exceptional antimicrobial properties and remarkable physical characteristics. This study reports the phytochemical screening of *Allium jacquemontii* plant extract for the preparation of AgNPs. The prepared nanoparticles (NPs) were characterized via UV- spectroscopy, X-ray diffraction analysis (XRD), scanning electron microscopy (SEM), Fourier-transform infrared (FTIR) spectroscopy, and transmission electron microscopy (TEM) and were evaluated for antimicrobial and pharmacological activities. HPLC analysis showed that *A. jacquemontii* had the lowest quantity of ferulic acid (10.3 ppm) and the highest concentration of chlorogenic acid (317.9 ppm). The prepared AgNPs exhibited excellent antifungal potential against *Aspergillus niger*, with maximum growth inhibition of 64.4% while strong antibacterial activity of 14.3 mm against *Escherichia coli* at a dose of 10 mg/ml was found. Similarly, the highest antiparasitic activity was 75.41 ± 1.16 against Promastigote and 71.13 ± 0.12 against Amastigote at 200 µg/mg. Inhibition of glucosidase, and amylase was observed, suggesting potential antidiabetic properties. The AgNPs were found to be biofriendly with red blood cells (RBCs) of a healthy human. This study indicates the potential of *A. jacquemontii* inflorescence derived AgNPs.

## Introduction

Nanotechnology is a quickly developing field and is primarily concerned with particles and structures of lower than 100 nm in diameter^1^^,^^2^. Nanotechnology creates nanoscale particles with special properties by fusing the principles of chemical and physical processes. Because of their unique size, structure, and orientation, nanoparticles can provide new or improved characteristics^3^. Specific properties like size, shape, and varying electrical, optical, theoretical, and magnetic aspects, for example, are of interest to nanoparticle researchers^3^. Nanoparticles (NP) have many applications including antimicrobial protection^4,5^ and drug delivery^6^ in the biomedical field. Nanotechnology is built upon the foundation of individual units and molecules that enable the creation of materials on a microscopic scale.

Nanoparticles (NPs) possess unique attributes, including size, distribution, and morphology that prove them extremely desirable for several applications (Yusuf, et al*.* 2023). Among the many kinds of metal-based NPs, AgNPs have received huge attention because of their effectiveness as antimicrobial agents, along with non-toxicity and the diverse range of applications both in the laboratory and in the living organisms^7–9^. AgNPs are also applied to treat wounds caused by bacteria. Another noteworthy application of silver nanoparticle gel or spray is in the pharmaceutical and cosmetics sectors for medicinal uses. AgNPs are also used in metals removal as photocatalytic agent^10,11^. There are many procedures for producing silver nanoparticles, like chemical, physical, photochemical, irradiative, and biological processes^12^. The physio-chemical techniques of producing AgNPs have some drawbacks, including the involvement of poisonous chemicals, high pressure, high temperatures, and the creation of perilous byproducts^13^. The green synthesis strategy makes use of ordinary chemicals that are connected to harmless chemical agents, such as polysaccharides, mixed valence polyoxometalates, and biological techniques^14^.

The plant-based preparation of AgNPs is very beneficial as the organic molecules found in the leaves extracts of suitable plantsnot only reduce Ag + ions to AgNPs, but also act as excellent capping agent^15^. Currently, scientists are concentrating on the bio- manufacturing of NPs using various metals like zinc, iron, silver, platinum, and gold^16^. Because of their benign nature and superior physio-chemical and biological properties, AgNPs are abundantly studied and used^17^. Because they are rich in phyto-active compounds, plants are applied to make a diverse medications against various disorders all over the world^18^. Due to the inherent bioactive chemicals found in a variety of botanical sources, the preparation of AgNPs via plant extracts has become a viable and environmentally acceptable method in recent years^19^. When compared to traditional chemical procedures, the application of plant extracts as stabilizing and plummeting agents for nanoparticle synthesis has advantages including lower ecological impact, biocompatible nature, and cost-efficiency^20^. Additionally, the characteristics and biological potential of the resultant NPs are influenced by the phytochemical constituents of plant extracts.

*Allium* (ornamental onion) is a large genus of Alliaceae, having 800 species and 15 subgenera^21^^[,22^. *Allium* is a rich source of phytonutrients and bioactive compounds (Pinela et al., 2017). It is used in folk medicine for the treatment of different diseases such as fevers, body pain, bites, cholera, blood pressure, and dysentery^23^. *Allium* species have strong antioxidant properties in all organs, especially in leaves and bulbs of wild and cultivated forms. Particularly high antioxidant concentrations are present in *Allium sativum (garlic),* including flavonoids and phenols^24^. *A. jacquemontii,* a lesser-known member of the Allium genus, has recently garnered scientific interest due to its diverse pharmacological properties, including antimicrobial, antidiabetic, and other biological activities^25^. Phytochemical analyses reveal the existence of flavonoids, saponins, alkaloids, and sulfur-containing compounds, which are believed to contribute to its bioactivity. Preliminary studies indicate significant antibacterial and antifungal effects, potentially linked to organosulfur compounds^26^. Moreover, ethanolic extracts of *A. jacquemontii* have demonstrated promising antidiabetic activity in vivo, showing notable reductions in blood glucose levels^27^. High-Performance Liquid Chromatography (HPLC) is a commonly used technique for the separation, identification, and quantification of bioactive compounds in complex plant extracts. With the progression of modern medication and pharmacological research, chemical synthesis has arisen as the primary method for producing medicinal agents in technologically advanced countries.

The purpose of existing study was to explore the synthesis of AgNPs through *A. jacquemontii* extract and assess their antibacterial, antifungal, antiparasitic, and pharmacological activity. To the best of our information this is the first study on the synthesis of AgNPs from *A. jacquemontii extract.* The study also focused on the phytochemical screening of the *A. jacquemontii* extract via HPLC for the effective preparation of AgNPs and utilized various techniques such as X-ray diffraction (XRD), UV spectroscopy, scanning electron microscopy (SEM), Fourier-transform infrared (FTIR) spectroscopy, and transmission electron microscopy (TEM) to analyze the synthesized nanoparticles. Additionally, the antimicrobial potential of the prepared NPs was assessed against and *Escherichia coli*, and *Aspergillus niger* while their antiparasitic effects were tested on Promastigote and Amastigote forms (the two morphological forms found in the life cycle of certain protozoan parasites, especially in the genus Leishmania, which causes leishmaniasis). The study also explored the AgNPs’ potential antidiabetic activity through glucosidase and amylase inhibition assays, as well as their biocompatibility with human red blood cells. Ultimately, this research aimed to assess the broader therapeutic potential of *Allium jacquemontii*-derived AgNPs for various pharmacological applications.

## Materials and methods

### Chemicals and equipments

The reagents used in this experiment required additional purification; they were all analytical grade and obtained from reliable sources like Fluke and Merck. Purity was ensured by preparing solutions with deionized water. Silver nitrate (AgNO_3_), methanol (CH_3_OH), Petri plates, test tubes, Whatman filter paper (No. 1), nutritional agar broth, and sodium hydroxide were the main chemicals and materials used in the experiment.

### Plant collection and extract preparation

*A. jacquemontii* at flowering stage were collected after the permission from the owner of the land during March–April and was identified by a well-known taxonomist (Dr. Moona Nazish) and deposited to the National harbarium (Voucher no. AK/2025–66) Quaid-I-Azam University, Islamabad, Pakistan. Plants were dried under shade at a temperature of 33ºC and a humidity of 50%. The shade was created using a shade net in room. The required temperature was acheived by selecting a site in a warm climate and humidity awas was maintained through steady air circulation using exhaust fan. The dried plant sample was then grinded into powered For the extract preparation the plant powder was mixed with deionized water with a ratio of 1:4 w/v, and stirred for 30 min through magentic stirror having 300 rpm. After filtration, supernatants were collected, and the water was allowed to evaporate in oven at 50ºC (overnight) to make the crude extract which was stored for experimental analysis.

### Phytochemical analysis

For the qualitative analysis of the different bioactive components, the method^28^ was used. For example, alkaloids, saponins, flavonoids, phenols, the extract were investigated by using simple phytochemical tests.

### HPLC analysis

In short, 5 mL of 10% methanol were used to dissolve 1 mg of plant extract, and 0.45 µm pore size membrane filters were used for filtering. The Agilent 1260 HPLC system with a reciprocating pump and C18 column (Sorbex RXC-8, dimensions 18 μm, 4.6 × 100 mm) was operated at 30 °C to identify phenolic acids. It works by drawing solvent into a cylinder and then pushing it through the column via a piston that moves back and forth. Additionally, a 0.45 µm pore size filter was used to filter the mobile phase. An ultrasonic bath was used to degas the mixture, and an eusion gradient containing 0.2% H_3_PO_4_, methanol, and acetonitrile was used to separate the mixture at a flow rate of 1 mL/min. After increasing the mobile phase by 5%, 50%, 70%, and 100% in 5, 15, 25, and 30 min, respectively, it was kept isocratic for the next five minutes. A wavelength adjustment of 210 nm was made for 35 min, and the autosampler was used to run standards and plant samples (5 µL) at various retention times. The K-factor was then calculated based on the retention time, and the calibration curve was plotted to estimate the concentration of each compound (ce.g. hlorogenic acid, hydroxybutyric acid, sinapic acid, ferulic acid) present in the plant extract. The “K ”value (retention) of the *A jacquemontii compounds* was calculated using the following formula:

1 $$K=(tR-tM)/tM$$

where t_R_ denotes retention time, and t_M_ is the dead time.

### Synthesis of the plant extract

For the preparation of the NPs 35 mg of the dried *A. jacquemontii* powder was mixed and homogenized in 130 mL of deionized water, and the mixture was incubated for 24 h in a shaking water bath. The shaking was performed at 100 rpm for 24 h to ensure thorough extraction of bioactive compounds. The solution was then filtered with the help of Whatman filter paper No. 1. The resultant filtrate solution was allowed to stand for two days. Then filtrate was kept standing for two days to allow for the complete extraction, stabilization, and settling of any fine particulate matter. This is generally done at room temperature (25 ºC) and in a dark to protect sensitive phytochemicals from light-induced degradation.

### Nanoparticles synthesis

AgNPs were prepared by a green synthesis method. Using a 10 mL graded cylinder, 1 mL of AgNO_3_ solution (99% purity, supplied by Shanghai Chemical Reagent Company) with a concentration of 0.01 M was first measured. It was then diluted up to 10 mL using deionized water, yielding a concentration of 0.001 M. NaOH (99.95% purity) purchased from Sigma-Aldrich was added to the mixture. The pH was tuned to 10 using this NaOH in the NPs preparation to create an alkaline condition that assists the reduction of silver ions (Ag⁺) to AgNPs and promotes the uniformity and stability of the NPs. The pH was then periodically re-verified through calibrated pH meter and adjusted as needed to maintain a stable pH of 10 throughout the synthesis. After adding 4 ml of centrifuged extract solution, the color of the solution changed to a pale brownish yellow, which is a sign that AgNPs were forming. The plant extract was centrifuged at 10,000 for 15 min prior to mixing.The *A. jacquemontii* extract contains phytochemicals like flavonoids, phenolic and phenolics that act as reducing agents in NPs formation.These compounds also stabilize the NPs by capping them and preventing aggregation. The pale brownish yellow paste was heated at 40 °C for 72 h. The NPs powder was obtained as a result of this strong heating. Prior to use in activities the prepared nanoparticles were characterized through spectroscopic and microscopic techniques.

### Characterization of the prepared nanoparticles

To study the optical properties, assessment of functional groups and surface configuration of the prepared NPs were characterized via FTIR (SPECTRUM, 65), UV (SPECORD 200 Plus, Analytik Jena, Germany), SEM (SEM, JEOLJSM 25,910), XRD (XRD, Bruker, D8), and TEM (JEOL JEM-2100 – Japan). The nanoparticle size was calculated via the Debye–Scherrer equation.

### Antimicrobial activity

#### Antibacterial assay

The preserved cultures of bacterial strains were obtained from the laboratory of Plant Pathology, Quaid-I-Azam University, Islamabad. The colonies were grown on nutrient media to obtain fresh cultures. The agar well diffusion procedure was used for the antibacterial assay of AgNPs. The antibacterial activity was assessed at various concentrations of AgNPs including 2.5 mg/mL, 5 mg/mL and 10 mg/mL with 75 µL of each concentrations was dropped into each well (having size of 6 mm) individually, while Carbapenem was used as a positive control and DMSO was used as a negative control. *Escherichia coli, Staphylococcus aureus, Salmonella typhi* and *Pseudomonas aeruginosa* were used. Each concentration was performed in triplicates. The ZOI was determine by measuring the bald through ruler. The antibacterial assay was executed using a previously published protocol^29^. The antibacterial activity was measured using the following formula:

2 $$\text{\%Inhibition}=\text{Ti}/\text{Ci}\times 100$$

whereT_i_ = inhibition in the test andC_i_ = inhibition in the control.

#### Antifungal assay

The pure preserved cultures of fungal strains were likewise obtained from the laboratory of Molecular Plant Pathology, Quaid-I-Azam University, Islamabad. The colonies were grown on potato dextrose medium (PDA) to obtain the fresh cultures. The antifungal activity was assessed at various concentrations of AgNPs including 2.5 mg/mL, 5 mg/mL and 10 mg/mL, while Fluconazole and DMSO were used as a positive and negative control, respectively. Each concentration was performed in triplicates. The ZOI was determine by measuring the bald through ruler. To determine the percentage antifungal potential; *Aspergillus niger*, *Verticillium dahliae*, *Candida albicans* and *Alternaria alternata* were used by following the standard methodology^30^, and the antifungal activity was quantified through the subsequent formula:

3 $$Growth{\kern 1pt} Inhibition\% = \times (C - T)/C$$

where C means the growth of the fungus in the control plate, and T shows to the growth of fungi in a AgNPs treated plate.

### Pharmacological applications

#### Antiparasitic assay

To determine the antiparasitic potential of the prepared AgNPs, they were applied against a parasite (*Leishmania* t*ropica promastigotes*), according to the previously documented protocol^31^. Briefly, various concentrations of AgNPs (from 25 to 200 g/mL) were applied on *L.* t*ropica promastigotes* parasites by the 3-(4,5-dimethylthiazol-2-yl)−2, 5-diphenyl-tetrazolium bromide (MTT) assay. The tested concentrations of the NPs were selected on the basis of previously reported research^8,9^ with a little modification. Amphotericin B was used as positive control. After the mixing of various doses of the prepared NPs into the wells of the microplates, incubation for 48 h at 27 °C followed. After that, 10 μL of MTT solution was added to each well and the microplates were then incubated for 4 h at 27 °C. The cell viability values were determined via a microplate reader (Fluoroskan Ascent, Thermo Labsystems, Helsinki, Finland) at 500 nm. *Leishmania* t*ropica promastigotes* were obtained from the Laboratory of Parasitology, Quaid-I-Azam University, Islamabad. The % inhibition was determined through the following formula (4):

4 $$\text{\% Inhibition}=100\times \text{Asample}/\text{Bcontrol}$$

where A_sample_ represents the AgNPs-treated sample absorbance and B_control_ means the absorbance of the control sample.

#### Antidiabetic assay

The antidiabetic efficacy of bio-fabricated AgNPs was examined by means of ɑ-amylase and α–glucosidase. A previously published procedure^32^ with slight changes was used.

Briefly, 10 μL of phosphate buffer, 30 μL of alpha-amylase, 20 μL of the AgNPs sample, and 30 μL of starch substrate were mixed. During this activity, various concentrations of the prepared AgNPs from 20 to 320 µg/mL were applied along with acarbose (CAS no. 56180–94-0) as a positive control. The microplate was then incubated for 30 min at 60 °C. Each well received a solution containing 20 mL of 1 M HCl and 90 mL of iodine solution after the incubation time. A microplate photometer was used to measure the absorbance at 520 nm.

To guarantee the stability and activity of the enzyme, α-glucosidase was dissolved in 50 mL of phosphate buffer solution (pH 6.5) containing 100 mg of bovine serum albumin (BSA). The reaction mixture consisted of phosphate buffer (pH 6.5), p-nitrophenyl-D-glucopyranoside, a substrate specific to α-glucosidase, and the test sample (AgNPs).. To start the process, the mixture was incubated at 35 °C for 15 min. After the first incubation, the reaction mixture was mixed with α-glucosidase enzyme solution, and it was incubated for an additional 10 min at 35 °C to encourage the enzyme to hydrolyze the substrate. A UV–Vis spectrophotometer was then used to test the reaction mixture’s absorbance at 400 nm. Reduced enzyme activity was demonstrated by elevated absorbance values, which is an indicator of inhibition.

The following formula was used to get the % inhibition (Abs = absorbance):

5 $$\text{\% Enzyme inhibition}=\left(\frac{\text{Abs Sample}-\text{Abs negative control}}{\text{Abs blank}-\text{Abs negative control}}\right)\times 100$$

#### Biocompatibility assay

A simple hemolytic test was to analyze the biocompatible nature of the prepared nanoparticles by treating freshly extracted human red blood cells following the former standard procedure^33^. Briefly, a health volunteer donated a blood sample of 2 mL in a tube. EDTA (ethylenediaminetetraacetic acid) was added to the blood-containing tube to avoid the clotting. Then the collected blood was centrifuged at 1200 rpm for 15 minsto separate the erythrocytes (red blood cells, RBC) from other components of the blood. The pellet was washed three times in PBS and the supernatant was discarded. The pellet was washed and mixed with 200 µl of isolated erythrocytes and 8.9 mL of PBS (pH: 7.3). 100 µl of erythrocyte suspension was mixed with 100 µl of various AgNPs (20-160 µg/mL)in Eppendorf tubes and incubated for 60 min at 30 °C and 10 min. Dimethyl sulfoxide was used a positive control. A microplate reader was used to calculate hemoglobin release absorption at 520 nm. The biocompatibility test was performed according to international standard guidelines after the approval from the departmental ethical committee of Quaid-I-Azam University Islamabad having reference no. BPS-2/2025. all methods were performed in accordance with the relevant guidelines and regulation. The percent hemolysis was calculated using the following formula (6).

6 $$\% {\kern 1pt} Hemolysis = (sample{\kern 1pt} Abs - neg.{\kern 1pt} {\kern 1pt} control{\kern 1pt} Abs)/(positive{\kern 1pt} Abs - neg.{\kern 1pt} {\kern 1pt} control{\kern 1pt} Abs) \times 100$$

### Statistical assessment

All the activities were performed in triplicates and SPSS, version 16.0 and OriginPro9 was applied to analyzed it statistically.

## Results and discussions

### Phytochemical analysis

The qualitative analysis showed the presence of secondary metabolites including phenols, flavonoids, alkaloids, saponins and tannins in abundance in deionized water extracts (Table 1). The findings of the present study revealed that phenols are the most abundant compounds followed by flavonoids^34^.

**Table 1:** Qualitative phytochemical analysis of *Allium jacquemontii* using distilled water (AJDW) solvents.

Sr. No	Metabolites	AJDW
1	Phenols	** + + + **
2	Flavonoids	** + + **
3	Alkaloids	** + + **
4	Saponins	** + + **
5	Tannin	** + **

Key: + + + : shows the presence of abundance, + + : shows the presence of moderate quantity, + : shows existence but in small concentrations.

### *Identification of fractions *via* HPLC*

**Fig. 1:**
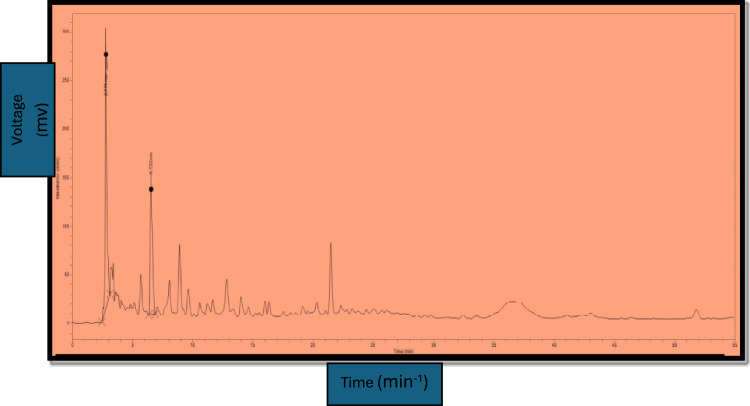
Chromatogram of HPLC for *Allium jacquemontii* extract.

**Fig. 2:**
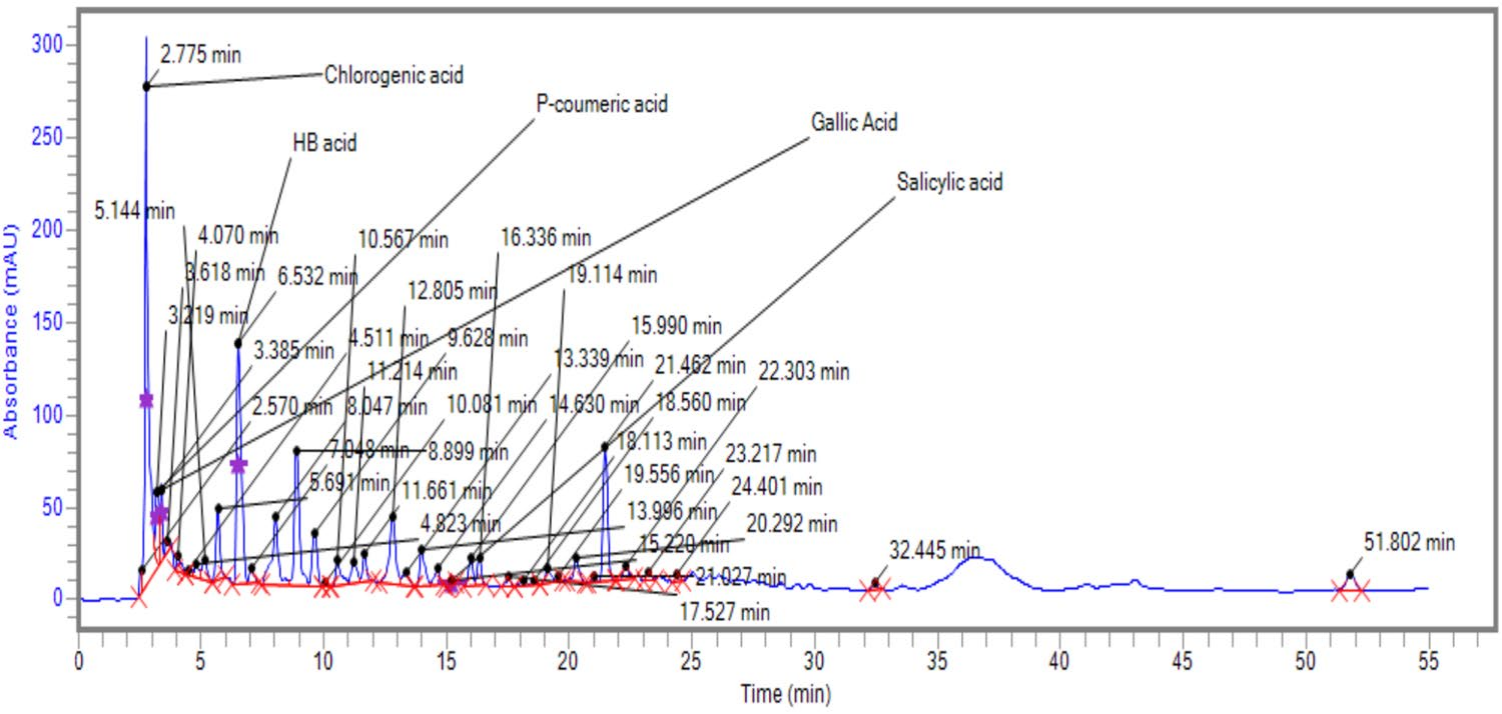
Peak allocation in the HPLC *Allium jacquemontii* extract spectrum. HB = hydroxybutyric.

HPLC plays a pivotal role in exploring the composition of natural products*.* The presence of some selected compounds in *A. jacquemontii* is shown in Figs. 1 and 2. *A. jacquemontii* extract was dissolved in 10% of aqueous methanol (i.e. water:methanol = 90:10 v/v) exhibited the highest concentration of chlorogenic acid (317.9 ppm) followed by hydroxybutyric acid (283.7 ppm), sinapic acid (45.3 ppm), vanillic acid (14.2 ppm) and ferulic acid (10.3 ppm) (Table 2). To determine the potential of *A jacquemontii* in the biomedical field, complete metabolic profiling of bioactive constituents is essential to assess the role of individual phenolic compounds and their associated properties. Various studies have reported the separation of phenolic acids (Kumar et al., 2017).

**Table 2:** Concentration of five phenolic compounds observed in *A. jacquemontii* using HPLC.

Compounds	RT when single standard was run	RT when mixture of standards was run	K-factor	Concentration (ppm)
Chlorogenic acid	2.775	2.880	0.00013	317.9
Hhydroxybutyric acid	6.532	6.759	0.00016	283.7
Sinapic acid	12.029	12.238	0.0000567	45.3
Vanillic acid	8.055	7.899	0.0000777	14.2
Ferulic acid	12.871	12.512	0.0000767	10.3

*RT: Retention time; ppm: parts per million.

Numerous bioactive chemicals found in medicinal plants are a blessing from nature^35^. According to^36^, phenolic acids reduce aging and related diseases like diabetes and cancer. Antioxidant, anti-ulcer, anti-pyretic, and neuroprotective properties are displayed by chlorogenic acid. According to^37^, it also aids in the treatment of diabetes and cardiovascular disorders and controls the metabolism of fat and glucose. Antibacterial, antioxidant, anti-cancer, anti-inflammatory, and anti-anxiety properties are demonstrated by sinapic acid. 4-Vinylsyringol, a decarboxylation derivative of sinapic acid, is a strong antimutagenic and antioxidative agent that inhibits the production of inflammatory cytokines and carcinogenesis. According to^38^, sinapine, also called sinapoyl choline, is thought to be an acetylcholinesterase inhibitor with potential therapeutic uses in the cure of a variety of illnesses. The biological actions of these substances have been reported to enhance the medicinal value of *A. jacquemontii*. Therefore, the current study recommends the isolation and characterization of these compounds.

### Characterization of Bio-fabricated AgNPs

#### UV–Vis Spectrophotometry of AgNPs

**Fig. 3:**
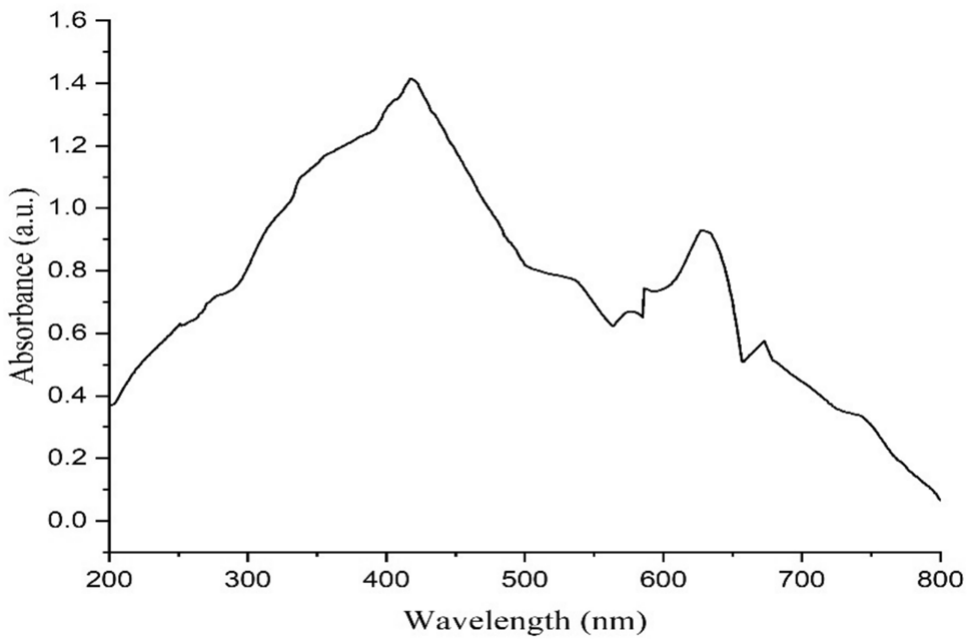
UV spectrum of plant mediated AgNPs, showing a maximum at 410 nm.

The optical behavior of the prepared AgNPs was scrutinized via UV–Vis spectroscopy in a range of 200 to 800 nm. The peak was developed at a wavelength of 410 nm (Fig. 3). The obtained peak value was closely aligned to previous work^39^. It has previously been proven that AgNPs show absorption at a comparable peak 400 nm^40^. This little variation may be due to the botanic source that has been used in the preparation of the NPs.

#### XRD study of AgNPs

**Fig. 4:**
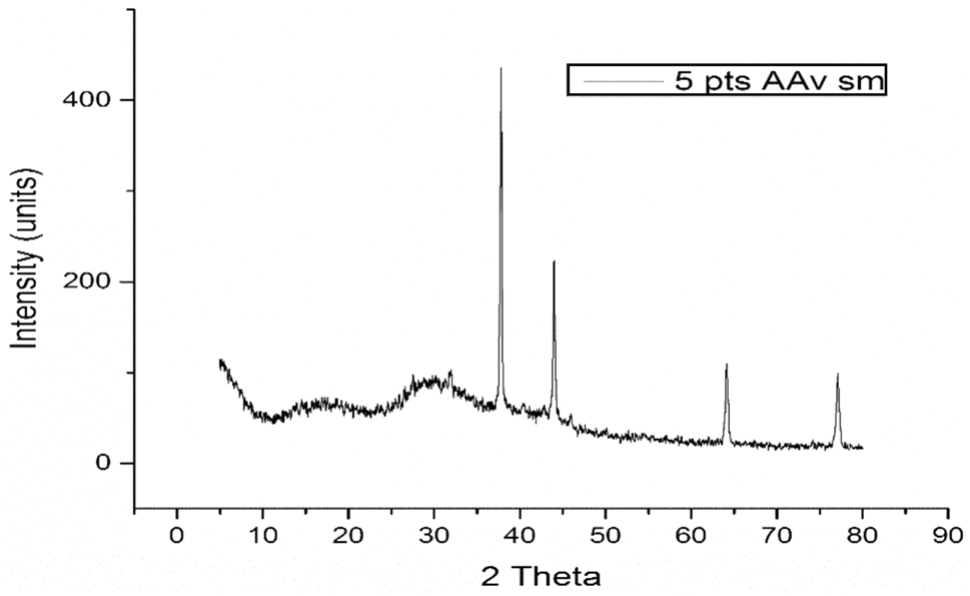
XRD spectrum of the prepared AgNPs.

The XRD was executed to explore the crystalline behavior of the prepared AgNPs. The crystalline properties of the NPs contribute vitally to their performance (Recio-Poo et al., 2023). The plant mediated NPs proved crystalline nature and showed characteristic peaks at 2θ. The XRD analysis revealed peaks at 2 θ values of 37.2 θ, 44.3 θ, 63.2θ and 77.2 θ which were indexed to (63), (130), (145), (160) and (165) planes of a cubic structure (Fig. 4.) following the JCPD card no. 04–0783. The strongest peak of AgNPs, established their crystalline nature. The XRD pattern of the AgNPs is aligned with former studies (Jaiswal et al., 2023). The particle size was calculated as 55 nm. The crystalline sizes of the prepared NPs were calculated with the help of the Debye–Scherrer equation as follows:

7 $$D=k\uplambda /(\beta cos\theta )$$

where D shows the crystalline size (nm), k is a constant, λ denotes the wavelength of the X-ray radiation, β specifies the full width at half maximum (FWHM) of the intensity and broad peaks and θ is the Bragg’s or diffraction angle.

#### FTIR study

**Fig. 5:**
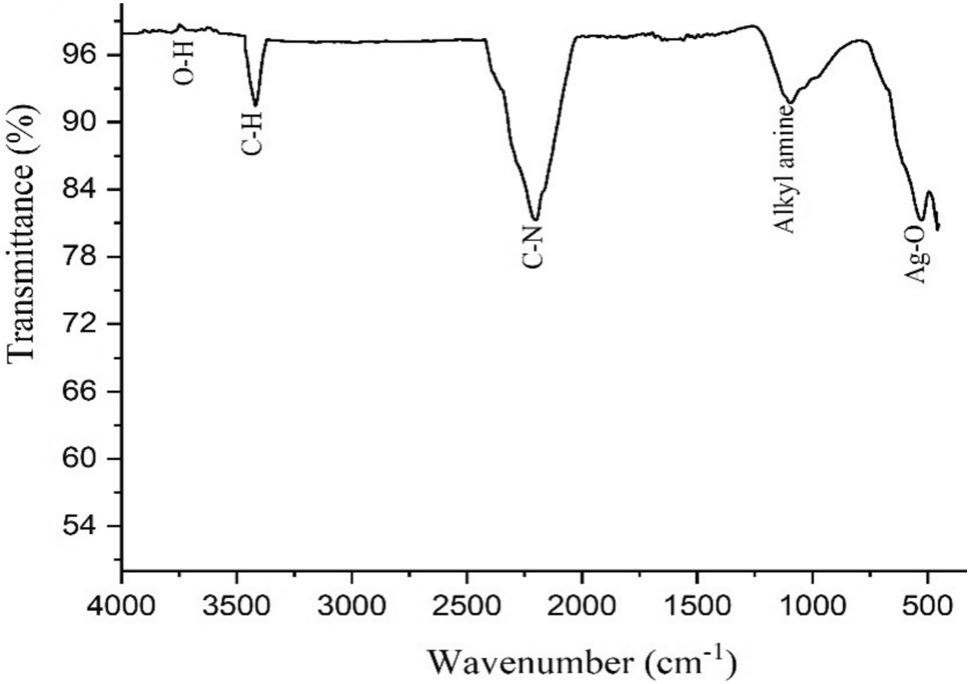
FTIR spectrum of phyto-fabricated AgNPs.

The FTIR pattern of the plant extract (Fig. 5) displays that the peak at 3600 cm^−1^ corresponds to the O–H bond. The peak present at 3450 cm^−1^ confirmed the presence the C–H bond of aromatic compounds while the peak found at 2200 cm^−1^ indicates stretching of the N–H bond. Similarly, the peak at 1000 cm^−1^ indicates the stretching of alkyl amine while the peak at 500 cm^−1^ represents Ag–O, which is close to the 519.92 cm^−1^ reported by^41^. The difference might be due to the botanic source and experimental conditions. These findings revealed that the phytochemicals present in the aqueous extract could have contributed in the reduction and the stabilization of AgNPs^42^. The obtained results are closely interlinked with previously published reports that acquired similar peaks for the green synthesized AgNPs^43^.

#### SEM analysis

**Fig. 6:**
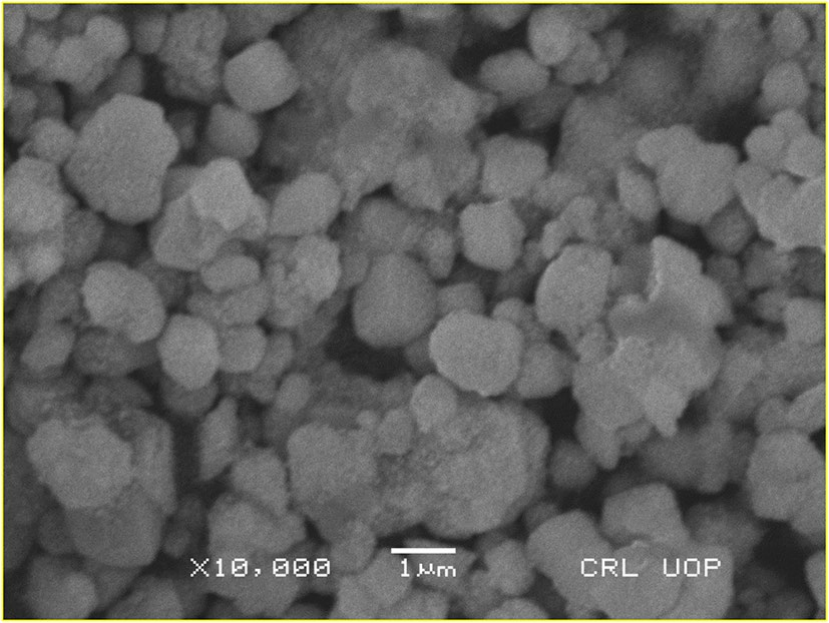
SEM image of bio-fabricated AgNPs.

Surface configuration of the prepared AgNPs was assessed via SEM. SEM investigation revealed the spherical shape of the NPs having a size from 55-60 nm in dispersed manner at 10,000X magnification (Fig. 6). These results are aligned with previously published work^44,45^.

#### Transmission electron microscope

**Fig. 7:**
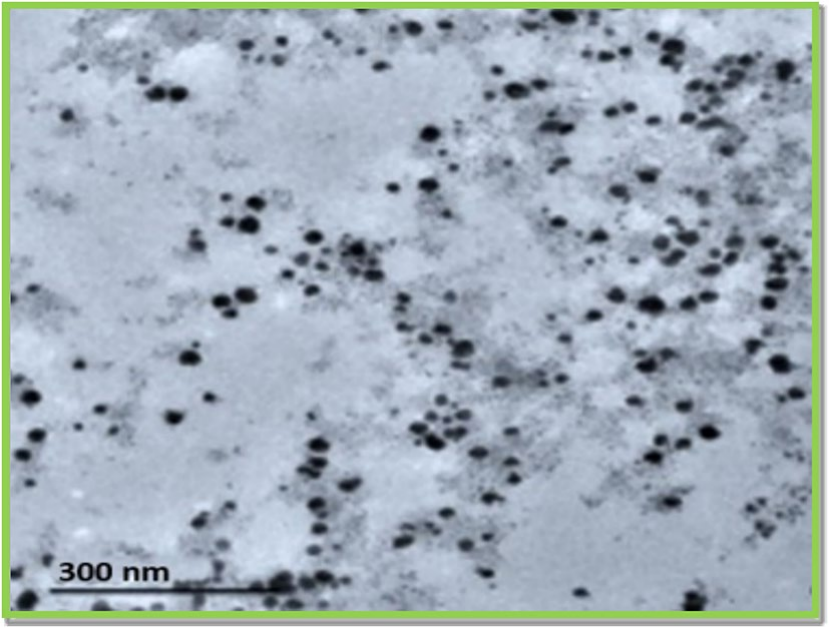
TEM image of the bio-fabricated AgNPs.

According to the Transmission Electron Microscopy findings, which are presented in Fig. 7. the prepared NPs are mostly of a spherical shape. All these findings are aligned with the earlier work^46^^,^^47^. The spherical shape and nanoscale dimensions of the AgNPs make them able for applications in catalysis, drug transportation, and biomedical activities^48^.

### Antimicrobial assays

#### Antibacterial activity

In the present study, *Pseudomonas aeruginosa, Salmonella typhi, Escherichia coli,* and *Staphylococcus aureus* were utilized for antibacterial activity testing. The antibacterial potential of the prepared NPs is shown Table 3. 100 µl of each AgNPs concentration (2.5, 5 and 10 mg/mL) was used to determine the inhibition zone (ZOI). The highest ZOI was 14.3 mm at 10 mg/mL, trailed by 13.1 mm at 5 mg/mL and 11.1 mm at 2.5 mg/mL against *E. coli* (Table 3), while the lowest ZOI was 14.0 mm at 10 mg/mL, followed by 11.6 mm at 5 mg/mL and 10 mm at 2.5 mg/mL against *S. aureus* (Table 3). In an antibacterial trial, the cell membrane is the first line of defense to protect the bacteria from the foreign agent. The larger surface to volume ratio of AgNPs causes the production of more reactive ions. These reactive ions lead to DNA damage, lysis of proteins, and breakdown of enzymes and finally causes the bacterial death^49^. Our findings are in good agreement with earlier related studies^50^^,^^17^^,^^44^.

**Table 3:** Antibacterial activity of prepared AgNPs as zones of inhibition (ZOI) for 75 µl samples.

S. No	Bacterial species	Positive control (in mm) (Carbapenems)	Negative control (DMSO)	Deionized water extract		
				Zone of Inhibition (mm)		
				2.5 mg/mL	5 mg/mL	10 mg/mL
1	Escherichia coli	20 mm	0 mm	11.1 mm	13.1	14.3 mm
2	Pseudomonas aeruginosa	21 mm	0 mm	10.2 mm	12.9	13.8 mm
3	Salmonella typhi	20 mm	0 mm	10.1 mm	12.4	13.5 mm
4	Staphylococcus aureus	19 mm	0 mm	10 mm	11.6	14.0 mm

#### Antifungal potential

The antifungal assay of the bio-manufactured AgNPs using PDA containing petri plates was studied. The highest antifungal assay of 64.4% was examined against *A. niger* at a concentration of 10 mg/ml, followed by 61.1% at 5 mg/ml and 55.0% at 2.5/ml mg 55.0% as presented in Table 4. AgNPs are more reactive and stable than other silver particles because of their greater surface area and smaller size^51^. Over the past ten years, mycologists have effectively employed AgNPs to slow the growth of various fungal pathogens, such as *Aspergillus niger*, *Candida albicans*, and *Fusarium graminearum*^52^. According to earlier research, AgNPs’ antifungal properties stem from the generation of reactive oxygen species (ROS). Additionally, it has been reported that nanomaterials exhibit antimicrobial activity due to their strong interactions with the microbial wall^53^. AgNPs lye cell walls of fungi by several mechanisms and also modify the cell membrane’s structure resulting in wall and plasma membrane damage (Alavi et al., 2019). The mechanism causes depolarization, fluidity, and permeability alteration resulting in damage to membrane and wall, peptides dislocations and impart oxidative stress^54^. As a result, released Ag^+^ ions they enter the cytoplasm, disrupt signaling pathway mechanisms and disrupt DNA, RNA, and enzymes by disturbing ribosomes assembly^55^.

**Table 4:** Antifungal activity of bio-manufactured AgNPs (2.5 mg/mL, 5 mg/mL & 10 mg/mL).

S. No	Fungal species	Positive control (in mm) (Fluconazole)	Negative control (DMSO)	AgNPs		
				**Zone of Inhibition (%)**		
				2.5 mg/mL	5 mg/mL	10 mg/mL
1	A. niger	70	0	55	60.1	64.4
2	A. alternata	69	0	50	55.4	61.2
3	C. albicans	69	0	50	54.4	61
4	V. dahlia	65	0	48.3	53	60.1

### Pharmacological activities

#### Anti-diabetic asssay

*Diabetes Mellitus* (DM) is considered to be inflicted by high glucose levels due to the inadequate insulin production by pancreatic cells. Lowering postprandial hyperglycemia, which can be accomplished through inhibiting Alpha-amylase (AA) and α–glucosidase (AG), which are vital glycan hydrolyzing enzymes in the gastrointestinal tract is one of the effective therapeutic approaches for *diabetes mellitus*^56^. In the present work, different doses of AgNPs ranging from 10 µg/mL to 320 µg/mL were analyzed for AA and AG inhibition as depicted in Table 5. The highest inhibition was 61.24 ± 0.35% calculated at the 320 μg/mL for AA whereas it was 59.56 ± 1.34% for α-glucosidase, correspondingly. Such noteworthy anti-diabetic activity of AgNPs against diabetes enzymes may lead to new pharmacological strategies to cure DM^57^.

**Table 5:** Alpha-amylase and α–glucosidase assay of plant-mediated AgNPs.

Conc. µg/mL	Alpha-amylase		α–glucosidase	
	AgNPs (%)	Acarbose (%)	AgNPs (%)	Acarbose (%)
320	61.24 ± 0.35	79.32 ± 1.33	59.56 ± 1.34	80.44 ± 1.83
160	45.14 ± 0.52	70.61 ± 1.62	45.27 ± 0.29	60.50 ± 1.76
80	35.41 ± 0.38	60.54 ± 1.18	34.59 ± 0.35	55.31 ± 1.22
40	25.13 ± 0.36	51.44 ± 0.84	22.44 ± 0.23	45.33 ± 1.34
20	20.13 ± 0.25	30.38 ± 0.77	18.42 ± 0.34	40.44 ± 0.68

#### Antileishmanial activity

Leishmaniasis is an extremely rare, spreadable tropical and subtropical infectious disease parasite. In 87 countries the disease is endemic and there are 1.6 to 3 million new cases worldwide every year, according to the WHO. Phlebotomus and Lutzomyia sandflies bite humans and spread an intracellular parasite that causes the disease. The uncontrollable spreading of the disease is likely due to an unsuitable vector and both reasonable and ineffective therapy. As indicated in Table 6, the promastigote and amastigote cells cultures of *L. tropica* were examined in our investigation utilizing the MTT assay and AgNPs formulations ranging from 25 to 200 µg/mL. The parasite’s promastigote and amastigote forms each had a significant death rate of 75.41 ± 1.16 and 71.13 ± 0.12 at 200 µg/mL, respectively, along with dose-dependent cytotoxicity. Previous researchers have also observed dose dependent Antileishmanial Activity (das Neves et al., 2024).

**Table 6:** Antileishmanial activity of bio-inspired AgNPs.

Concentration (µg/m)	Promastigote inhibition	Amastigote	IC50 (µg/ml)	IC50 (µg/ml)
200	75.41 ± 1.16	71.13 ± 0.12	77.43 Amastigote	68.33 Promastigote
100	65.44 ± 0.23	61.11 ± 1.09		
50	55.14 ± 1.20	50.33 ± 1.14		
25	39.22 ± 0.26	38.39 ± 0.10		

#### Biocompatibility assay

The hemolytic assay was performed to analyses the toxic nature of the phyto-bombarded NPs against the RBCs. RBC and AgNP concentrations from 20 to 160 μg/mL were combinedly cultured in a buffer solution prepared to imitate an extracellular environment. The hemolytic assay based on the release of hemoglobin due to the lysis of the RBCs when AgNPs are applied. The findings of the hemolytic activity are presented in Table 7**.** The American Society for Testing and Materials documented that the substance with hemolysis > 2% are non-hemolytic^58^. It can be examined from the findings (Table 7) that bio fabricated AgNPs revealed biocompatibility even at the highest dose. These results make them able for biomedical usage. Our results are aligned with the previous work and the prepared AgNPs be subjected to beneficial applications^59^,. The prepared NPs interacts with cell membranes to influence physiological activities and supports immunity, aiding in autoimmune^2^.This integrative approach supports safer and more effective biomedical applications of AgNPs when combined with traditional medicine^60,61^.

**Table 7:** % Hemolytic activity of bio-fabricated AgNPs.

**S.NO**	**Concentration (µg/mL)**	**Hemolysis %**	Positive control (Dimethyl sulfoxide)
1	160	1.33 ± 0.11	4.33 ± 0.12
2	80	0.86 ± 0.14	4.33 ± 0.12
3	40	0.69 ± 0.10	4.33 ± 0.12
4	20	0.50 ± 0.13	4.33 ± 0.12

## Conclusion

This study demonstrates the promising potential of silver nanoparticles (AgNPs) synthesized using *Allium jacquemontii* plant extract, highlighting their remarkable antimicrobial, antiparasitic, and antidiabetic activities. Comprehensive characterization of the synthesized AgNPs via UV spectroscopy, XRD, FTIR, SEM, and TEM confirmed their successful production and provided insights into their structural and physical properties. The AgNPs exhibited significant antifungal activity, particularly against *Aspergillus niger*, and displayed strong antibacterial effects against *Escherichia coli*. Additionally, they showed potent antiparasitic activity against both promastigote and amastigote forms, as well as notable inhibitory effects on glucosidase and amylase enzymes, indicating their potential as antidiabetic agents. Importantly, the AgNPs demonstrated biocompatibility with human red blood cells, further supporting their safety for biomedical applications. Overall, the findings of this study underscore the therapeutic potential of *Allium jacquemontii*-derived AgNPs in various pharmacological applications, paving the way for future research into their clinical utility as novel antimicrobial, antiparasitic, and antidiabetic agents.

## Data Availability

The data is available on the request from the corresponding author.
